# Assessing transparency and accountability of national action plans on antimicrobial resistance in 15 African countries

**DOI:** 10.1186/s13756-021-01040-4

**Published:** 2022-01-24

**Authors:** Anne Harant

**Affiliations:** 1 Faculty of Business, Economics and Social Sciences, University of Hamburg, Max-Brauer Allee 60, 22767 Hamburg, Germany; 2 German Institute of Global and Area Studies (GIGA), Neuer Jungfernstieg 21, 20354 Hamburg, Germany

**Keywords:** National action plans, Transparency, Accountability, Africa, LMICs, Health policy

## Abstract

**Background:**

Antimicrobial resistance (AMR) poses an increasing public health threat to low- and lower-middle income countries. Recent studies found that in fact poor governance and transparency correlate more strongly with AMR than factors such as antibiotic use. While many African countries now have national action plans (NAPs) on AMR, it is unclear whether information is publicly available on their implementation, surveillance and financing.

**Methods:**

Here, the transparency of information related to AMR national action plans in 15 African countries is assessed, based on a governance framework for AMR action plans. Public availability is assessed for AMR documents, progress reports, AMR surveillance data, budget allocations, as well as bodies and persons responsible for implementation of NAPs. Government websites and search engines were perused using search terms related to the studied criteria and countries.

**Results:**

Results show that most countries have a national action plan publicly available. AMR surveillance data was available for a few countries, but systematic progress reports and funding allocations were absent in all but one country. Information on a body mandated to coordinate NAP implementation was available for most countries, but their functionality remain unclear. Most countries have nominated at least one person responsible for AMR nationally. In general, information was often fragmented and frequently available on external, non-government websites. It appears that commitments on AMR made in the often comprehensive NAPs are rarely met in a timely manner, exhibiting rather weak accountability for AMR results. The article provides concrete policy recommendations on how transparency and accountability may be improved with little effort.

**Conclusions:**

Making information available can enable stakeholders such as civil society to demand accountability for results and lead to much needed specific actions on curbing AMR in countries.

**Supplementary Information:**

The online version contains supplementary material available at 10.1186/s13756-021-01040-4.

## Background

Antimicrobial resistance (AMR) is deemed one of the central public health challenges of the twenty-first century. The former United Nations director general Ban Ki-moon called AMR “a fundamental, long-term threat to human health, sustainable food production and development. […] Without AMR containment, the Sustainable Development Goals for 2030, such as ending poverty, ending hunger, ensuring healthy lives, reducing inequality, and revitalizing global partnerships are unlikely to be achieved.” [1]. Policies to curb resistance have increasingly been launched to tackle the cross-border problem of AMR on a global scale [2–4]. While AMR has leveled off in some high-income countries, it continues to rise in low- and middle-income countries (LMICs) [5]. Evidence of successful and comprehensive national AMR policies and programs in LMICs to date is scarce [6–9]. This is not least due to fragmented health systems in many low- and middle-income countries, which often lack sufficient governance mechanisms and financial resources. Recent studies found that in fact poor governance and corruption correlates more strongly with antibiotic resistance than antibiotic use and other commonly assumed drivers of AMR [10, 11]. The lack of control, enforcement and oversight of policies and regulations in the distribution of antimicrobials as well as perverse financial incentives for prescribers are only some of the factors contributing to this problem [12–14]. For many African countries, reviews found significant levels of resistance to commonly prescribed antibiotics [15, 16] and high rates of AMR in food animals [17]. At the same time, availability and quality of data on AMR in many African countries is low [15] and AMR preparedness in sub-Saharan African countries has been found to be rather low [18].

To tackle AMR globally through a holistic and multi-sectoral One Health approach, the Food and Agriculture Organization of the UN, the World Organization for Animal Health, and the World Health Organization (WHO) formed a tripartite collaboration. This tripartite body advises countries on how to develop a national action plan (NAP) on AMR and has established a NAP database for monitoring progress on a global scale [19]. The global action plan on AMR urges all WHO Member States to have national action plans on AMR in place that are aligned with the global action plan [20].

In 2015 the WHO found that only 34 out of 133 participating in a survey reported on having a NAP to tackle AMR [21] Since then the number of countries with a NAP has been increasing, to 117 (from 159 responding) in 2018/2019 and 119 (from 135 responding) in 2019/2020 [22]. However the database also confirms what the Interagency Coordination Group on AMR states in its discussion paper on AMR NAPs, that “in most countries, the greatest challenge is not writing a NAP but implementing it and demonstrating sustained action.” [23]. The Lancet Infectious disease commission on AMR found that, so far, progress on national action plans has been episodic and uneven [5]. Particularly in low- and middle-income countries, the implementation of NAPs is often weak or stalling [7, 14, 21]. Long-term funding, the sustained focus of national leaders and country-level actors, as well as harmonized monitoring indicators are urgently needed [5].

In the absence of effective national government accountability, the implementation of NAPs is unlikely to progress significantly and fast enough on a global scale. Being held accountable for meeting the policy commitments written down in the AMR NAPs is essential to achieving sustained funding and action on AMR. One current theory of change stipulates that transparency, by informing stakeholders, facilitates their participation in policy making and thereby enables accountability [24, 25]. Interested stakeholders, such as civil society, can use publicly available information for external scrutiny and hold governments to account by demanding action to meet their commitments. Another important component of accountability are sanctions, compensations or remediation, should standards or commitments not been met [26, 27]. To that end, the WHO policy package on AMR stresses that a well-informed public is a catalyst to health actions and building strong public awareness is vital [28]. It advises to develop an accountability framework on AMR and for civil society representatives to be involved in the development of AMR policies as well as their implementation and monitoring. Improving transparency to enable external scrutiny and demands for action can be essential in advancing measures on AMR. An accountability framework is particularly important in the African region, where the AMR problem is significant and requires immediate and sustained action and where implementation is currently rather weak, as the Tripartite data as well as local experts suggest [22, 29].

Assessments of governance, transparency and accountability in the health and pharmaceutical sector have been put forward in recent years to identify vulnerabilities, gaps as well as strengths in health systems and encourage governments to make more information publicly available [30, 31]. Anderson et al. have developed a governance framework for the assessment of AMR NAPs to support their sustained implementation [32]. The framework identifies transparency and accountability as two of the central governance domains. So far, the framework has not been operationalized and applied for a multi-country assessment of transparency and accountability in Africa.

This paper offers the first operationalization of Anderson et al.’s framework to assess public availability of key information on AMR NAPs. It further provides the first comprehensive overview of key information on AMR NAPs in Africa, which can inform interested stakeholders such as policy makers and civil society and help build capacity in countries. It is hoped that this study encourages countries to prioritize sharing timely information on AMR NAPs in the future, to facilitate sustained action against AMR.

## Methodology

The objective of this research is to assess transparency and accountability aspects of AMR NAP implementation in 15 African countries.

### Country selection

For this study, African countries have been selected if they met the following criteria: (1) have English as an official language and also (2) responded to having developed a national action plan in the self-assessment questionnaire submitted to the tripartite AMR database in 2018/2019, i.e. answering C, D or E in response to item 5.1 “Country progress with development of a national action plan on AMR”. The countries included were: Ethiopia, Ghana, Kenya, Liberia, Malawi, Mauritius, Namibia, Nigeria, Seychelles, Sierra Leone, South Africa, Tanzania, Uganda, Zambia and Zimbabwe.

### Assessment criteria

Assessment criteria were selected and adapted from the NAP governance assessment framework by Anderson et al. [32], specifically from panel 1, domain four (accountability) and domain five (transparency). For each of the selected countries, it has been assessed whether the following information, presented in Table 1, is publicly available. For the purpose of this study, information was considered publicly available if it could be found on a publicly accessible website, either as grey literature or peer reviewed literature. In Table 1, each of the six criteria have been listed (column 1) and operationalized for measurement, specifying three response options “Yes,” “Partly” and “No”.

**Table 1:** Response options for assessment criteria

Assessment criteria			Response options		
Type of information		Corresponding domain in Anderson et al. governance framework	Yes	Partly	No
1	National action plan on AMR	Transparency (domain five)	NAP document on publicly accessible website	Parts of the NAP document publicly available	Document not publicly available
2	NAP AMR annual progress report(s)		Report available detailing implementation status and progress regarding objectives of the NAP	Fragmented information available in articles or reports on specific aspects of NAP implementation status (e.g. in MOH news articles or Bulletins)	No information on progress publicly available
3	AMR surveillance data		At least some AMR data available in GLASS report and/or through other national annual surveillance reports/databases	Information about surveillance system indicators reported to GLASS or surveillance data available but not for all years since reporting started	No AMR surveillance data publicly available
4	Funding allocated to AMR NAP		Information on government and potentially other funding allocated to AMR NAP implementation publicly available (e.g. in federal budget books or on other government websites)	Information on amount of at least some external funding for AMR NAP implementation available but no government budget	No information on funding publicly available
5	Name and structure of body with mandate to coordinate and implement NAP	Accountability (domain four)	Name of body and mandate publicly available	Name of body publicly available but mandate unclear or only plans to establish body but no information that is has been established	No responsible body with official mandate named publicly
6	Name of responsible person nominated in each sector		Name of responsible person nominated in each sector publicly available (human, animal and environment health)	Name publicly available of at least one responsible person nominated for specific sector, as general AMR focal point or as GLASS focal person	No name of any responsible individual publicly available

In the accountability domain, Anderson et al. (2019) also ask whether agreements exist regarding what happens if objectives are not met. This aspect has not been included in this study as a recent study of AMR NAPs in South East Asia found that “implications of unmet objectives were absent” [33]. If present at all, it is assumed that they may exist internally but are unlikely to be publicly available. With regard to public availability of surveillance data, AMR surveillance data have been included here, since standardized and recent monitoring data from the WHO Global Antimicrobial Resistance Surveillance System (GLASS) system with common indicators is available for 2019/2020 for numerous countries. Antibiotic consumption data has only been published once for 2016–2018 by the WHO, but no recent data has been released since has therefore been excluded from the assessment at this point.

### Search strategy

Searches were conducted in English between July and November 2020. Two types of searches were performed using keywords and synonyms representing the assessment criteria. During the first search, the relevant One Health ministries were identified for each country (usually Ministry of Health, ministry of agriculture and ministry of animal welfare; for a list of all ministries identified by country see Additional file 1). The websites of all identified government bodies were searched, using the internal site search function (if available), as well as google site search. Keywords and synonyms representing the assessment criteria have been used for an online search. For funding allocations, the federal budget for 2018, 2019 and 2020 from the ministry of finance or treasury websites were reviewed for allocations to the AMR NAP for each country.

Secondly, a google search was performed for each country and assessment criteria. The search engine google claims over 95% search engine market share in Africa [34]. For each search, results from the first two pages were reviewed for relevant links, as these generally receive more than 80% of the clicks [35] and interested stakeholders looking for information are generally not expected to go beyond the second page of google results. For each assessment criteria, various combinations of key words were used. For example, when looking for the NAP of Ghana, the following google search was carried out: Ghana (“antimicrobial resistance” OR “antibiotic resistance” OR “AMR”) (NAP OR “national action plan”). Additionally a snowball technique was used to identify further relevant information mentioned in documents or articles found through the searches explained above [36].

## Results

The public availability of information varied across assessment criteria and countries. While a NAP document was available for most countries (11/15), other information was only found in a fragmented manner (progress reports) or hardly at all (funding allocation). And overview of all results across assessment criteria and countries is presented in Table 2; more specific results are available in Tables 3 and 4 below. In the following, results for each of the assessment criteria will be elaborated.

**Table 2:** Results of assessment criteria for each country

Country	Information publicly available?					
	NAP	Progress reports	Surveillance data AMR	Funding allocation	Responsible body	Responsible person per sector
Ethiopia	Yes	Partly	Yes	No	Yes	Partly
Ghana	Yes	Partly	No	No	Yes	Partly
Kenya	Yes	Partly	Partly	No	Yes	Partly
Liberia	Partly	No	Partly	No	Yes	Partly
Malawi	No	Partly	Partly	No	Yes	Partly
Mauritius	Yes	Partly	Partly	No	Yes	Partly
Namibia	No	Partly	No	No	No	No
Nigeria	Yes	Partly	Yes	Yes	Yes	Partly
Seychelles	No	No	No	No	No	No
Sierra Leone	Yes	No	No	No	Partly	No
South Africa	Yes	Yes	Yes	No	Yes	Partly
Tanzania	Yes	Partly	Partly	No	Yes	Partly
Uganda	Yes	Partly	Yes	No	Yes	Partly
Zambia	Yes	Partly	Partly	No	Yes	Partly
Zimbabwe	Yes	No	Partly	No	Yes	Partly

### National action plans

For 11 of the 15 countries a NAP was publicly available. The comprehensiveness, lengths, structure and content of plans was very diverse. Some included targets and costing (e.g. Ghana), others were more general and announced the development of implementation plans and budgeting in further documents (e.g. Ethiopia). South Africa, for example, has produced the NAP in two separate documents, the “National strategy framework” and an implementation plan. Ethiopia has a “Strategy for the prevention and containment of AMR”, which can be found in the WHO library of national AMR action plans. However, the implementation action plan announced therein to follow as a separate document has not been found as a result of this search. Similar to Ethiopia, some countries used other titles than national action plan for their documents, such as “national strategic plan” (Sierra Leone) and “national strategy framework” (South Africa), which may complicate finding this information for interested stakeholders. Table 3 consolidates all titles of the NAPs found as well as links to the documents, where available.

**Table 3:** Titles of NAPs and links to documents

Country	Title of NAP	Link
Ethiopia	Strategy for the prevention and containment of AMR for Ethiopia 2015–2020	http://www.fmhaca.gov.et/wp-content/uploads/2019/03/Strategy-for-the-Prevention-and-Containment-of-AMR-in-Ethiopia-Oct-2015.pdf
Ghana	Ghana national action plan on antimicrobial resistance	http://www.moh.gov.gh/wp-content/uploads/2018/04/NAP_FINAL_PDF_A4_19.03.2018-SIGNED-1.pdf
Kenya	National action plan on prevention and containment of antimicrobial resistance	http://www.health.go.ke/wp-content/uploads/2018/02/Kenya-NAP-6th-Nov-2017-3.pdf
Liberia	*Developed and validated, but not yet published*	
Malawi	National AMR strategy (2017–2022) that incorporates an action plan has been developed, but not publicly available	National AMR strategy; launched 2018 but not publicly available
Mauritius	Republic of Mauritius National Action Plan on Antimicrobial Resistance 2017–2021	http://www.fao.org/faolex/results/details/en/c/LEX-FAOC171515/; https://www.who.int/antimicrobial-resistance/national-action-plans/library/en/
Namibia	Information that final draft of NAP is awaiting high-level approval	http://siapsprogram.org/publication/containing-antimicrobial-resistance-through-rational-antimicrobial-use-in-namibia/
Nigeria	National action plan for antimicrobial resistance 2017–2022	https://ncdc.gov.ng/themes/common/docs/protocols/77_1511368219.pdf
Seychelles		
Sierra Leone	National strategic plan for combating antimicrobial resistance 2018–2022	http://apps.who.int/datacol/answer_upload.asp?survey_id=666&view_id=722&question_id=13163&answer_id=19958&respondent_id=278587
South Africa	South African antimicrobial resistance national strategy framework; a one health approach 2018–2024	http://www.health.gov.za/index.php/antimicrobial-resistance
Tanzania	The national action plan on antimicrobial resistance 2017–2022	https://www.mifugouvuvi.go.tz/uploads/publications/en1526401722-NATIONALACTIONPLANFNL10May2017(1).pdf
Uganda	Antimicrobial Resistance National Action Plan 2018–2023	https://cddep.org/blog/posts/uganda-amr-nap/
Zambia	Multi-sectoral National Action Plan on Antimicrobial Resistance 2017–2027	https://www.afro.who.int/publications/multi-sectoral-national-action-plan-antimicrobial-resistance-2017-2027
Zimbabwe	Zimbabwe One Health antimicrobial resistance national action plan 2017–2021	https://www.ed.ac.uk/files/atoms/files/zimbabwe_nap_2_1.pdf

Some NAPs could not be found, such as that of Namibia, which seems to be waiting for approval since 2018. According to the Fleming Fund, which supports LMICs to generate, share and use AMR data, Malawi has developed a NAP, published as the “National AMR Strategy” [37] and screenshots showing the front and content page of the Liberia national action plan were found, but the actual documents were not available online. The WHO Joint External Evaluation of Malawi from February 2019 notes inadequate dissemination of the AMR strategy and plan at all levels to ensure stakeholders are aware of its content and of their roles and responsibilities [38], reiterating the importance of dissemination following publication to increase transparency.

### Progress reports

No written national annual progress reports have been identified that systematically address progress of the NAP for any country. South Africa has made annual PowerPoint progress reports available, in which progress on different aspects of the AMR strategy is detailed [39]. For most of the other countries however, at best fragmented information on specific implementation activities in diverse formats has been found (see additional file for links to information found for each country). For example, an AMR surveillance implementation report in Ethiopia (available for 2018 but not 2019); scientific assessments of aspects of NAP implementation in Ghana [40] and Tanzania [41]); articles by health ministries or institutes on AMR activities, e.g. in Kenya and Zambia; and updates in the epidemiological bulletin in Nigeria; and an agenda for an Online Workshop on Implementation Status and Reprioritization of Zimbabwe’s One Health Antimicrobial Resistance National Action Plan.

The most comprehensive external progress reports found were the Medicines, Technologies, and Pharmaceutical services (MTaPS) Program annual and quarter reports, available for Ethiopia, Kenya, Tanzania and Uganda [42]. However, the reports are limited to activities financed by the US Agency for International Development (USAID) for the respective country and are not systematically reporting on all aspects of the NAPs. They provide progress updates on activities such as “Strengthening MSC governance structures and functions” and mention if activities, due in the respective quarter, had not been addressed as planned and why (in 2020 this was primarily because COVID-19 activities took preference).

For several countries, information such as new AMR guidelines were found, that had been developed during the NAP implementation timespan. These findings indicate that progress is indeed happening, but is not systematically communicated. Other articles explicitly state that implementation is stalling, as for example in Namibia where “due to resource issues and competing priorities, the implementation of [the Namibian] action plan has faced challenges” [43].

### AMR surveillance data

Four countries have reported AMR surveillance data in the WHO GLASS report 2019/2020 for all or most of the GLASS pathogen indicators [44]. Six countries have reported information on national surveillance system indicators, but no actual surveillance data. Ghana is enrolled but has not reported any information and Malawi had reported surveillance data in 2017/2018 but none in 2019/2020. In the WHO JEE assessment report for Malawi it is mentioned that an annual AMR report is shared amongst stakeholders, but this could not be found online [38]. For most countries there are further single scientific studies available that measure resistance levels for various pathogens, which are however vertical in nature and not linked or integrated into a national system.

The Fleming fund notes in one of its reports that there is little perceived use of surveillance data on any level and also low demand for that data from policy makers [45]. It can be assumed that this is true in many countries and suggests that surveillance data should be shared much more actively amongst various stakeholders to foster its use for evidence-based policy making.

### Information about funding allocations

Information about funds allocated to AMR activities were hardly available, particularly with regard to government budget. An official allocation of federal budget was only found for Nigeria, labelled as “Implementation of National Action Plan for antimicrobial response in Nigeria” and “Sustaining Antimicrobial Resistance surveillance in sentinel sites in Nigeria” [46]. While several countries have costed their NAP, the sources of funding are either not specified or rather broad, listing e.g. NGOs, General Operating Grants or external donors. In several NAPs, for example in Malawi, a shortage of resources has been identified as a risk to a successful implementation. In its Request for Proposal for Zimbabwe, the Fleming Fund (a UK Aid programme that supports countries to collect, analyse, share and use high-quality data on antimicrobial resistance) acknowledges that although the NAP is costed, it is unlikely that the budget allocations will match the aspirations, which limits implementation [45]. Nine countries currently receive grants from the Fleming fund and several other AMR-funding sources, such as the Global Antibiotic Resistance Partnership (GARP) or the AMR Multi-Partner Trust Fund. However, such information about donor funding is primarily available from donor websites, not national governments.

It could be hypothesized that the lacking availability of information on funding allocations means that no funding is dedicated to AMR activities. However, for several countries specific AMR related activities have been reported, such as guidelines development, workshops and surveillance activities. For example, in the “Guidelines for the prudent use of antimicrobials in animals” in Kenya, it is stated that these had been made possible through the Standards and Market Access Program (SMAP) of the Ministry of Agriculture, Livestock, Fisheries and Irrigation with support from the European Union (Guidelines). However, no AMR allocations could be found in the federal budget. It therefore seems that national budgets so far do not provide sufficient detail to identify allocations to AMR.

### Responsible bodies

For all countries that have a NAP publicly available, the name of at least one governance body responsible for tasks related to the NAP could be ascertained. The names of these bodies differ between countries (for overview see Table 4). The responsibilities of the bodies vary across countries and not all have explicitly been tasked to coordinate and implement the NAP, sometimes the responsibilities are shared between two different bodies. An overview of all names and responsibilities is provided in Table 3. In some NAPs governance structures and relations between different ministries and stakeholders are displayed, but no explicit information has been found publicly on whether the AMR bodies are officially accountable to the government, as demanded in the Anderson et al. framework [32].

**Table 4:** Results on responsible bodies and responsible persons nominated

Country	Responsible body		Responsible person(s)	
	Name of body	Responsibilities	Responsibility	Affiliation
Ethiopia	National Advisory Committee on Antimicrobial Resistance Prevention and Containment (NACARC)^1^	Advise, guide, and harness the fight against the challenges of AMR in Ethiopia	Two GLASS focal persons	–
Ghana	AMR Secretariat	Coordinate AMR issues in the country	AMR focal point	Head of Public Health and Food Safety
Kenya	National Antimicrobial Stewardship Interagency Committee (NASIC)	The highest policy and governance body responsible for all AMR activities	Two AMR focal points	Ministry of Health/Ministry of Agriculture, Livestock and Fisheries,
	Technical committee	Responsible for implementation of the NAP, coordinating with stakeholders, and advising on guidelines, rules and regulations	Focal person for laboratory AMR surveillance and GLASS 2020 focal person	National Public Health Laboratory
			AMR focal point/Team lead AMR	Pharmacy and Poison board
Liberia	AMR Technical Working Group	–	AMR focal person	–
			AMR National focal point and GLASS 2020 focal person	Ministry of Health
Malawi	AMR National Coordinating Committee (AMRNCC)	The MoH, through the AMRNCC, manages AMR activities and sets agenda for AMR surveillance	National AMR focal point	National Microbiology Reference Laboratory
Mauritius	Steering Committee of the National Action Plan on Antimicrobial Resistance of the Republic of Mauritius	Has oversight of and is responsible for the development, review, implementation, monitoring and evaluation of the Mauritian NAP on AMR	National focal person	Ministry of Health
			GLASS 2020 focal person	Director Public Health, Ministry of Health and Wellness
Namibia	–	–	–	–
Nigeria	National AMR Steering Committee (NSC)	Oversee AMR-related activities within all sectors, plan and set direction, facilitate communication across members and groups, monitor progress	AMR focal lead	Federal Ministry of Agricultural and Rural Development
			Two GLASS 2020 focal persons	*Nigeria Centre for Disease Control*
Seychelles	–	–	–	–
Sierra Leone	AMR national multi			
sectoral coordinating group (NMCG)	–	–	–	
South Africa	National Ministerial Advisory Committee (MAC) for AMR	Mandated to coordinate intersectoral efforts nationally, provide advocacy and awareness and monitoring and evaluation of the implementation of the NAP	AMR Laboratory and Culture Collection-National Lead and GLASS 2020	National Institute for Communicable Diseases
Tanzania	National Multi-Sectoral Coordinating Committee (MCC) on AMR	Central National steering body and shall oversee and coordinate all AMR related activities. The AMR Action plan operations shall be managed and implemented through the MCC	AMR coordinator/focal point	Ministry of health, Community, Gender, Elderley and Children
			AMR focal point	Ministry of Livestock and Fisheries
			GLASS 2020 focal person	–
Uganda	National AMR Sub-Committee (NAMRsC)	Oversee and coordinate implementation of the NAP	GLASS 2020 focal person	
Zambia	National Multi-Sectoral Steering Committee (NMSC)	Guide, oversee and monitor AMR-related activities in all sectors	AMR focal point Human health	Lusaka District Health Office
	Antimicrobial resistance coordinating committee (AMRCC)	Coordinate and implement AMR related activities	AMR focal point Animal Health	Ministry of Fisheries and Livestock
			National AMR focal point and coordinator laboratory systems	Zambia National Public Health Institute
Zimbabwe	MoHCC secretary for health	Leading national AMR activities	National AMR focal point	Ministry of Health and Child Care
	AMR Focal point	Lead, coordinate activties of the NAP, facilitate and oversee implementation	AMR focal point for animal health	Central Veterinary Laboratory

^**1**^A recent MTaPS report suggests that the committee has been renamed into National Antimicrobial Resistance and Containment Advisory Committee (NAMRAC) [50]

The names and responsibilities have been mostly retrieved from the NAPs. While the plan to establish these bodies is mentioned in most NAPs, it is unclear in which countries these bodies have actually been formally established. It is also unknown whether they are meeting regularly, since Terms of Reference and meeting minutes do not seem publicly available. Limited information has been found indicating that the establishment of AMR bodies has been delayed in at least some countries. For example, a notice of application for appointment to the ministerial advisory committee on AMR in South Africa posted in late 2019 suggests that the MAC has not come into force before 2020; that is 5 years after the initial AMR NAP had been published with the aim to establish the committee. In a job opening in 2020 looking for an Antimicrobial Resistance Project Coordinator for Sierra Leona, a listed task was to “Lead and support the establishment and strengthening of the national multi-sectoral coordinating group (NMCG) for AMR”, suggesting that the committee has not been officially established yet [47]. In Nigeria an inaugural meeting of the AMR Coordination Committee has been reported to have taken place in 2020 [48]. For some countries, the above-mentioned MTaPS quarterly reports include short minutes of meetings on AMR, summarizing what has been discussed and including date, time and number of participants. These indicate that multi-sectoral AMR meetings have been taking place in Ethiopia, Kenya, Tanzania and Uganda [49].

Some NAPs included implementation matrixes listing outputs and the body responsible for each. However, very often numerous bodies were mentioned for each output as well as heterogeneous group such as “academia”, somewhat thwarting the idea of responsibility.

### Focal points

No country seems to have (publicly) nominated one responsible person for each of the three sectors, human health, animals and environmental health (see Table 4; further information on the names of nominated persons and links to data sources can be found in the Additional file 1). For Kenya, Tanzania, Zambia and Zimbabwe, two responsible individuals affiliated with ministries from two different sectors were nominated as AMR focal point. Only in Zambia, an explicit nomination for different sectors (human health and animal health) was identified, also detailed in Table 4. Most countries have nominated an overarching national AMR focal point. Other terms used were e.g. AMR focal lead (Nigeria), AMR Coordinator (Tanzania), AMR lead (South Africa); and team lead or AMR national focal person (Liberia). It is not exactly clear if the task and responsibilities are the same for all these positions. Departments at ministries have also been labelled “focal points” in various documents.

Some of the information on nominated individuals was found in the NAPs or associated documents. Other information could only be retrieved from e.g. participant lists in meeting agendas. For some countries, the only name available was that of nominated GLASS focal persons from the GLASS Report 2020. For the GLASS reports, a short questionnaire is sent annually to AMR national focal points and lists their names for each contributing countries under “collecting and compiling data for this report” [44]. The GLASS focal persons were found to be the same as otherwise nationally nominated AMR focal points only in South Africa, Zambia and Zimbabwe. In other countries the national focal persons where not the same as the GLASS focal persons.

### Overarching findings

Several observations were made during this study that are likely to impede accessibility, when interested stakeholders are searching for NAP information online. Across government websites, there was no subpage dedicated specifically to AMR. If documents were available from public websites, they were often hidden, for example in a list of documents in the download section or an article in the news section. Another observation was that the search function on several government websites did not always yield results on AMR, but applying the google site search option (e.g. “Antimicrobial resistance” site:[gov.website]) did find information on AMR on the same website. It can be assumed that most citizens are unaware of how to use this function. This could lead stakeholders to assume that no NAP or progress exists and discourage them from actively demanding information. Another observation was that documents were often not available from official websites of the national government, but rather from other organizations such as funding bodies or health organizations. Contact details for national focal points were usually not available, making it difficult for stakeholders to know from whom they can request information. In summary, the following steps are considered useful to improve transparency and accountability with regard to AMR NAPs:

- Consistent use of terms in line with global action plan on AMR
- Improve search functions of government websites
- Include publication date on articles, documents and postings
- Transparent and proactive reporting on progress as well as difficulties

Furthermore, it seems key to dedicate a subpage on a government website to consolidate all AMR information. This would show government ownership of information and could immensely increase transparency and enable stakeholders to consult both commitments and goals in the NAP as well as progress on those goals and commitments. Table 5 lists information that is considered useful to consolidate in one place for each country.

**Table 5:** Information to include on AMR section on government website(s)

National Action Plan on AMR (all versions)
Progress reports (at least annually)
Individual responsible person/focal point for AMR with full name and contact details
Outline AMR allocations in federal budgets, external funding, funding gaps and executed budget
Surveillance reports
Terms of reference for AMR NAP committees
Meeting minutes of AMR NAP committees
Agreements on what happens when NAP objectives are not met

## Discussion

The findings presented in this study are considered timely and policy relevant as they suggest that not only transparency, but also progress on AMR NAP implementation seems to be precariously lagging in many countries and accountability is urgently needed to ensure that NAP commitments are met in the future. This is particularly important considering the current preference given to resources for COVID-19 containment, which could further exacerbate already insufficient funding and political will on AMR. However, AMR is a more permanent and potentially much more severe pandemic, which can be slowed through better transparency and accountability.

It is important to reiterate that this study is looking at the public availability of information online. Even in cases where no information was found, this information might exist at government level internally, in hard copy format or in national languages. It should also be acknowledged that in many of the studied countries, the process to develop and implement policies tackling AMR have only commenced in recent years. Furthermore, the potential impact of COVID-19 pandemic on AMR related work has to be kept in mind. In many countries, the resources redirected towards the COVID-19 response will have aggravated problems to secure financing of AMR activities and hindered progress. It can be hoped that more progress can be observed in the coming years, such as development of surveillance systems as well as data collection and sharing thereof.

Anderson et al. ask to apply caution when using the framework for cross-country comparisons since the quality of documents and measures might differ significantly and ticking the same box for all countries does not mean the same quality or results [32]. It should be reiterated however, that this assessment does not appraise quality of measures, but ascertains whether key information on AMR NAPs is in the public domain. For example, it is not assessed whether the measures proposed in the NAPs are evidence-based or how well they are implemented. A progress report, which explicitly outlines that no progress has been made on NAP objectives, is still considered transparent. Anderson et al. note that, while their framework seems possibly ambitious for countries with limited resources, consulted experts from low- and middle-income countries confirmed the relevance of the framework criteria also for settings with fewer resources. It can be argued that the transparency and accountability aspects are in fact of particular importance for low- and middle-income countries to strengthen implementation of NAPs in face of limited resources.

### Comparison with responses to tripartite database

The Tripartite results for the 2019/2020 survey were published while this study was conducted and they show some discrepancies with the findings presented here [22]. Table 6 shows the responding countries and their responses to indicator 5.1 “Country progress with development of a national action plan on AMR” in both Surveys. Not all changes in responses since the 2017/2018 from countries studied in this article are in line with the findings of this study. For example, South Africa changed its response to a previous level from D (NAP approved with operational plan and monitoring arrangements) to C (NAP developed). This is somewhat in contrast to the information that has been found about implementation of the plan in recent years, which suggest that response “E” would be warranted. Ghana, for which a study has identified financing as a major hurdle for NAP implementation [40], and less information on implementation has been found here, has selected the most advanced response option “E”, indicating that funding sources have been identified, the NAP is being implemented and monitoring and evaluation in place. The multiple components of the response options make them ambiguous and difficult to compare across countries. The unambiguous response options bear the risk that some countries go for the lower response options, even if not all components of the response are in place, while others choose a response category if at least one of the components is fulfilled. This could explain the discrepancy between the tripartite data and the results presented here. Another possibility is that governments have essential information evidencing progress (or setbacks), which is not publicly available. The results in this study therefore indicate that the responses of the tripartite self-assessment may be interpreted and used with some caution. It could be valuable if countries provided additional information, such as documents or links (e.g. to progress reports, surveillance data, budgets) to substantiate their responses, where useful.

**Table 6:** Response to item 5.1 from tripartite survey

Response options to 5.1 “Country progress with development of a national action plan on AMR”		Country responses to tripartite surveys	
Letter	Description of response options	2017/2018	2019/2020
C	National AMR action plan developed	Namibia, Seychelles	South Africa
D	National AMR action plan approved by government that reflects Global Action Plan objectives, with an operational plan and monitoring arrangements	Ghana, Liberia, Malawi, Mauritius, Nigeria, Sierra Leone, South Africa, Uganda, Zimbabwe	Ethiopia, Kenya, Nigeria, Sierra Leone, Zambia
E	National AMR action plan has funding sources identified, is being implemented and has relevant sectors involved with a defined monitoring and evaluation process in place	Ethiopia, Kenya, Tanzania, Zambia	Ghana, Liberia, Tanzania

### Lacking budget information

The difficulties to find health budget line item information are in accordance with results from a recent study which found that national budgets did not include enough detail on Ministry of Health budget allocations to identify immunization [51]. Griffiths et al. further revealed inconsistency in inclusion of donor funding in budgets, and a general lack of budget execution reports (which show how allocations have actually been spent) as the greatest hindrance for budget transparency. A study of European NAPs also revealed scarce availability of identified funding sources, let alone detailed budget information [52]. In several cases, funding can probably not uniquely be attributed to AMR, such as funding of laboratories for disease monitoring. However, it would be possible to publicly outline from which funding stream or ministerial budget the money for a particular activity, such as AMR surveillance, is derived. Making AMR allocations, executed budgets as well as explicit funding gaps publicly available would significantly increase accountability. Importantly, it would help to understand whether actual costs correspond with initial costings of NAPs to help with future planning and budgeting. Publishing budget details will also give governments an opportunity to take credit for funding invested into AMR activities, so far often hidden in already existing programmes and budgets (e.g. for laboratories performing surveillance activities).

### What countries can gain through transparency on AMR activities

Good standards and formats for transparency on AMR have yet to be developed. Countries taking the lead now can set good examples and inspire others to follow suit. Just as pathogens, transparency can be contagious. Establishing transparency is often a rather low hanging fruit, as it merely requires making information publicly available that already exists at government level. In Zambia, for example, a workshop was held where participants were updated on the AMR National Action Plan implementation status [53]. Since this information is already available and consolidated, it would only be a small step for governments to also publish them on a suitable website and a great opportunity to show progress and inspire other countries to take action.

It can be assumed that many governments shy away from making available information on something that has not been achieved or is behind schedule. However, accountability also means explaining the reasons for not meeting commitments, which strengthens public trust in governments. This trust in turn is essential to the successful implementation of AMR activities, such as doctors accepting new practices of prescribing and patients following advice on taking antibiotics. Transparency can also enable and inspire participation and may activate stakeholders that want to advance the process and apply scrutiny in a productive way. One example is the Roll Back Antimicrobial Resistance Initiative in Tanzania, with Erick Venant as a local champion, that helps to implement the national AMR agenda and has called for the acceleration of the NAP implementation [54]. A recent paper reports on other promising examples of civil society as catalysts for action on AMR [55].

Similarly, international organizations and donors can contribute to strengthening transparency. For example, the WHO library for AMR NAPs as well as the FAOLEX database of the Food and Agriculture Organization are currently lacking several existing NAPs and could be updated more regularly to include all current versions. Donors that finance AMR NAP development and implementation could tie their funding to transparency clauses and require the public disclosure of e.g. annual progress reports and all funding allocations.

### Discrepancy between words and actions

In summary, the findings of this study reflect conclusions from the Interagency Working Group on AMR and others: particularly in LMICs, the biggest challenge is not formulating action plans, but implementing them [23, 56, 57]. The World Bank diagnosed a significant ‘doing’ gap with regard to AMR policy implementation [58]. This reiterates that a detailed NAP is no measure of success in itself and needs to be complemented by further information. A study looking at whether national action plans of eight LMICs were in line with international guidelines found that the NAPs of Ghana and Uganda were particularly comprehensive and included, amongst others, costing and funding sources [59]. But the authors emphasize that formulation of policies is not equal to implementation [59, 60]. For example, despite its great level of detail and completeness, the implementation of the NAP in Ghana is lagging [40] and in Tanzania an analysis found limited implementation of the NAP despite its launch 3 years prior “with all the necessary emphasis and a promise to act fast and efficiently” [41]. Mirfin Mpundu, director of the Africa chapter of the AMR network ReAct, summarized: “The problem is […] that African countries are not yet implementing to the full scale that is proportionate to the challenge we face. And I think that scares me.” [61], minute 3:04–3:15].

While there is no one-fit-all solution that can be easily applied, the Interagency Working Group on AMR also emphasizes the importance of strengthening transparency and accountability to close the implementation gap [57]. Khan et al. have studied barriers to implementing NAPs in LMICs and their results suggest that local context, including power dynamics and systemic constraints, require more focus [56]. They also identified conflicts of interests as barriers, since stakes connecting policy makers with the pharmaceutical and livestock industries can hinder implementation of harder regulatory approaches. This is an interesting finding as it reflects an issue that has been identified as problematic across the pharmaceutical sector and could be alleviated through increased transparency and conflict of interest policies [30].

An open discussion about the current approach in many LMICs would be helpful, which is that often donors fund the development of elaborate and ambitious NAPs. However, what is their use if these NAPs are then sitting on the shelves and are only very partially implemented? More focused plans could help to achieve accountability for a limited number of prioritized commitments. The University of Glasgow has launched a novel research project in Zambia to make prioritization an essential component of controlling AMR in LMICs. Through a workshop with all sectors involved Zambia has already produced a document prioritizing activities of their NAP in 2019 [62]. It remains to be see whether this prioritization exercise can facilitate NAP implementation.

## Conclusions

The results of this study showed that most countries have published their AMR NAPs and the number of countries sharing their surveillance data on AMR is slowly increasing. However, transparency on progress and funding allocations of AMR is very limited. It appears that governments rarely meet commitments on AMR made in the often comprehensive and detailed action plans, exhibiting rather weak accountability for AMR results. Increasing transparency on progress—or reasons for lack thereof—would strengthen accountability and enable stakeholders such as civil society to demand much needed specific actions on curbing AMR in countries around the world.

## Supplementary Information

**Additional file 1:** Additional information on results and websites searched.

## Data Availability

All data generated or analysed during this study are included in this published article and its additional information file.

## Abbreviations

AMR: Antimicrobial resistance
GLASS: Global antimicrobial resistance surveillance system
LMICs: Low- and middle-income countries
NAP: National action plan
MTaPS: Medicines, technologies, and pharmaceutical services
USAID: US Agency for International Development
WHO: World Health Organization
